# SiO_2_ Coated on ZnO Nanorod Arrays With UV-Durable Superhydrophobicity and Highly Transmittance on Glass

**DOI:** 10.3389/fchem.2020.00101

**Published:** 2020-02-20

**Authors:** Hong Li, Xinyan Zou, Hongyan Wei, Qiang Li, Qiang Gao, Qinzhuang Liu, Jinfeng Zhang

**Affiliations:** ^1^School of Physics and Electronic Information, Huaibei Normal University, Huaibei, China; ^2^School Key Laboratory of Green and Precise Synthetic Chemistry and Applications, Ministry of Education, Huaibei Normal University, Huaibei, China; ^3^Information College, Huaibei Normal University, Huaibei, China

**Keywords:** ZnO nanorod arrays, superhydrophobicity, solar cell, PLD, UV durability

## Abstract

ZnO nanorod arrays were fabricated on glass through hydrothermal way. Then a thin SiO_2_ film was covered on ZnO nanorod arrays using pulsed laser deposition (PLD) technique, and modified by stearic acid. It was found that SiO_2_ film only had slight effects on the contact angle and transmittance of ZnO nanorod arrays. However, it had brought a huge improvement in the UV durability of superhydrophobic ZnO nanorod arrays. The results showed that the water contact angle remains constantly at 160.5° even UV irradiation time exceeded 50 h when the deposition time of PLD was about 10 min. This structure with UV-durable superhydrophobicity and highly transmittance on glass substrate can be served as front materials in solar cells.

## Introduction

Superhydrophobic surfaces have won considerable attentions because of its important application in self-cleaning, corrosion resistance, microfluidic systems and so on (Maghsoudi et al., 2019; Qu et al., 2019; Lv et al., 2020; Zhu et al., 2020). Rough micro-nano surface and lower surface energy are responsible for the formation of superhydrophobic surface (Shchukin et al., 2006; Wang et al., 2015; Velayi and Norouzbeigi, 2018). Many methods have been adopted to manufacture superhydrophobic surface (Darmanin and Guittard, 2011; Li and Guo, 2019; Zhang et al., 2020). ZnO is a common material used to prepare superhydrophobic surface. The reason is that it is not only rich in raw materials but also the morphology is easy to adjust through various methods (Zang, 2018; Aydemir et al., 2019; Wang et al., 2019).

As dust accumulates on the glass surface, the efficiency of solar cells will be reduced by nearly half (Elminir et al., 2006). It is of great importance to design a self-cleaning surface for solar cells. In addition to this property, prepared film must have good transmittance in the range of sunlight as well as being UV-durable (Park et al., 2011). Nanostructure ZnO has high visible transmittance due to its high band gap. Compared with TiO_2_, the electron mobility in ZnO is higher, which can reduce the electron transfer time in the film (de Jongh and Vanmaekelbergh, 1996). In addition, ZnO and its doping have been widely used in solar cells (Shen et al., 2019), pressure-sensitive devices (Chen et al., 2016), and transparent conductive electrodes (Sharmma et al., 2017) due to its excellent photoelectric performance. Gao et al. (2011) fabricated highly transparent and superhydrophobic ZnO nanorod arrays on glass. It was found that this film had minimal impact on solar cell. However, the superhydrophobic surfaces formed by ZnO micro-nano structure are easy to lose their superhydrophobicity under sunlight because of photooxidative ability (Li et al., 2013). The wettability switch under UV irradiation from superhydrophobicity to superhydrophilicity on the surface of ZnO is caused by photochemical reactions (Feng et al., 2004).

SiO_2_ is a material of ceramics with excellent electrical insulation performance (Wang et al., 2009). Furthermore, SiO_2_ can easily form cross-linked chains and react with hydroxyl groups on the surface of ZnO (Al-Asbahi, 2017). Therefore, SiO_2_ can be acted as UV-resistant material to form UV-durable superhydrophobicity. It is reported that the SiO_2_ is covered on ZnO nanorod arrays to form UV-durable superhydrophobic surface (Li et al., 2019). SiO_2_ film can be prepared through various techniques (Xue et al., 2013). Compared to these techniques, pulsed laser deposition (PLD) is a superior method for the growth of oxide film and it has many advantages, such as high deposition rate, low substrate temperature, precise stoichiometry, easily controlled thickness and so on (Nikov et al., 2019). SiO_x_ thin layer was fabricated on the surface of isolated multi-walled carbon nanotubes through PLD and the thickness can be précised controlled (Ikuno et al., 2003). SiO_2_ thin film was prepared on different substrate in the case of O_2_. It was found that the transmittance of the 400-nm-thick films was 95% at a 500 nm wavelength (Okosh et al., 2002).

SiO_2_ films were deposited on ZnO nanorod arrays through PLD method and modified by stearic acid. The results showed that superhydrophobicity of SiO_2_/ZnO/glass remained stable even after prolonged UV irradiation. In addition, the light transmittance reached 85% in the visible region. Thus, the SiO_2_/ZnO/glass with superhydrophobicity and high transmittance may provide an effective application in solar cells.

## Experiments

### Preparation of ZnO Seed Layer on Glass

C_4_H_6_O_4_Zn·2H_2_O was dissolved in ethanol at room temperature. The concentration and volume of C_4_H_6_O_4_Zn·2H_2_O solution was 0.005 M and 100 ml, respectively. The solution was stirred for 2 h to form a stable suspension. The length, width and thickness of the Corning 7059 glass were 4 cm, 4 cm, and 1 mm, respectively. Glasses were sequentially cleaned by hydrochloric acid, acetone before being dried at room temperature. The solution was dropped onto the surface of glasses by straw and waited for the ethanol to evaporate. This process was repeated for six times. Finally glasses were annealed in muffle furnace at 350°C for 30 min.

### Preparation of ZnO Nanorod Arrays

Three glass substrates were suspended vertically in three sealed beakers which containing 8 mM zinc nitrate hydrate 200 ml and 8 mM hexamethylenetetramine 200 ml. The three bakers were heated at 95°C in furnace with 2, 4, and 6 h, respectively. The glass substrates were then rinsed with deionized water and dried in the air.

### Deposition of SiO_2_ Film

ZnO nanorod arrays were coated by SiO_2_ film through PLD with λ = 248 nm KrF. The base pressure was 10^−4^ Pa. During the preparation of the sample, the O_2_ was introduced into the chamber. The pulse frequency, operating pressure and laser energy density were 5 Hz, 5 Pa, and 1.8 J/cm^2^, respectively. The deposition time was 5 and 10 min. The distance between the silica dioxide target and the substrate was 45 cm.

### Hydrophobization of Samples With Stearic Acid

Both SiO_2_ coated and uncoated ZnO nanorod arrays were immersed in ethanol solution of stearic acid (8 mM, C_18_H_36_O_2_) 100 ml for about 24 h at room temperature.

### Characterization of Sample

Surface morphology, diffraction signal and water contact angle were performed by field-emission scanning electron microscope (SEM, JEOL, JSM-6610LV), X-ray diffractometer (XRD, XDAL-3000), High resolution transmission electron microscope (HRTEM, JEOL, JEM-2100) and contact angle measuring instrument (JC2000C1). The surface composition and photoluminescence spectrum of films were carried by Fourier transform infrared (FTIR, FP-6500) spectrometer and spectrofluorometer (FLS920 325 nm). The transmittance of the films was measured by ultraviolet-vis spectrometer (UV-Vis, Lambra-950). The ultraviolet light was provided by the 300 W mercury lamp (CEL-LAM300, 50 mW cm^−2^, maximum light intensity at 365 nm). The distance between the light source and the sample was 30 cm.

## Results and Discussion

**Figure 1:**
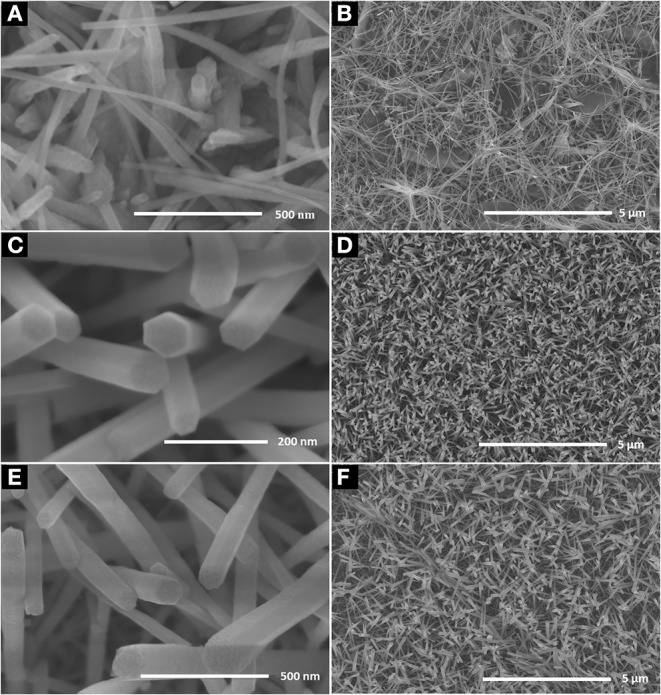
SEM images of the ZnO nanorod arrays coated on glass substrate with different reaction time **(A)** 2 h, **(C)** 4 h, and **(E)** 6 h; **(B,D,F)** are as-prepared large area ZnO nanorod arrays of the corresponding samples.

**Figure 2:**
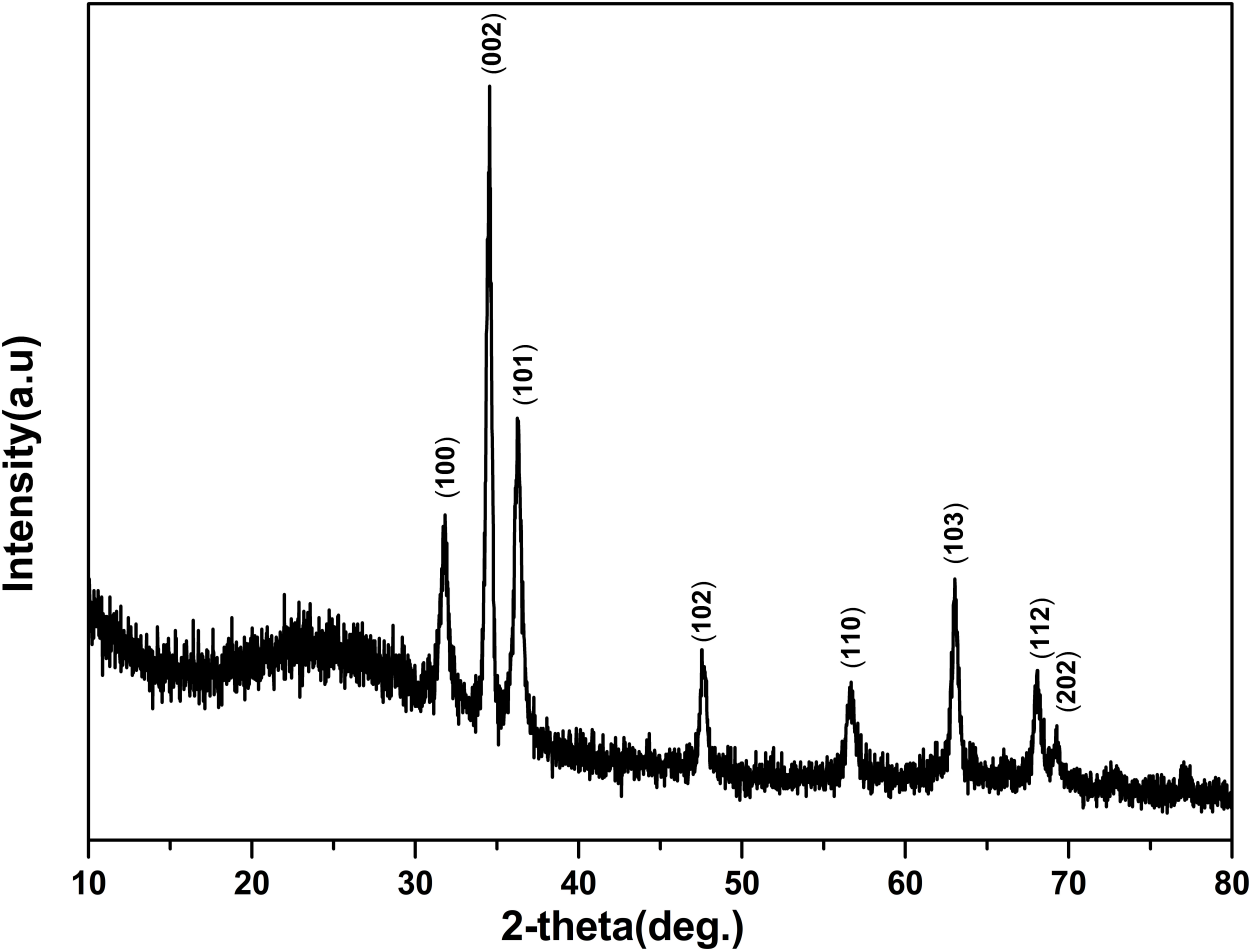
X-ray diffraction patterns of the ZnO nanorod arrays coated on glass substrate with reaction times 4 h.

Figure 1 shows the SEM images of ZnO nanorod arrays prepared on glass with different reaction times. When reaction time is 2 h (Figures 1A,B), the diameter of ZnO nanorod is small, and some ZnO nanorod entangled with each other. It is shown in Figures 1C,D, the diameter is bigger and about 40–60 nm when reaction time is 4 h. in addition, ZnO nanorod stood nearly perpendicularly on the glass and the number of ZnO nanorod is about 36 roots μm^−2^. With the increase of reaction time, the diameter and number of ZnO nanorod is about 150 nm and 10 roots μm^−2^, respectively. The X-ray diffraction patterns of ZnO nanorod arrays with reaction time of 4 h were shown in Figure 2. Compared with the peaks of (100), (101), (102), (110), (103), (112), and (202), the intensity of (002) peak is much stronger than that of the others, showing that the ZnO nanorod arrays are c-axis oriented. In the formation of ZnO nanostructure under hydrothermal conditions, the growth velocities are $V_{\left[0001\right]}\;>\;V_{\left[01\bar{1}0\right]}>V_{\left[000\bar{1}\right]}$, resulting in the formation of six-prism ZnO nanorod (Liu et al., 2009). As reaction time increases, ZnO nanorod exhibits increased diameter. In addition, their surfaces will become a little rougher due to dissolution. This can be confirmed in the Figures 1E,F.

**Figure 3:**
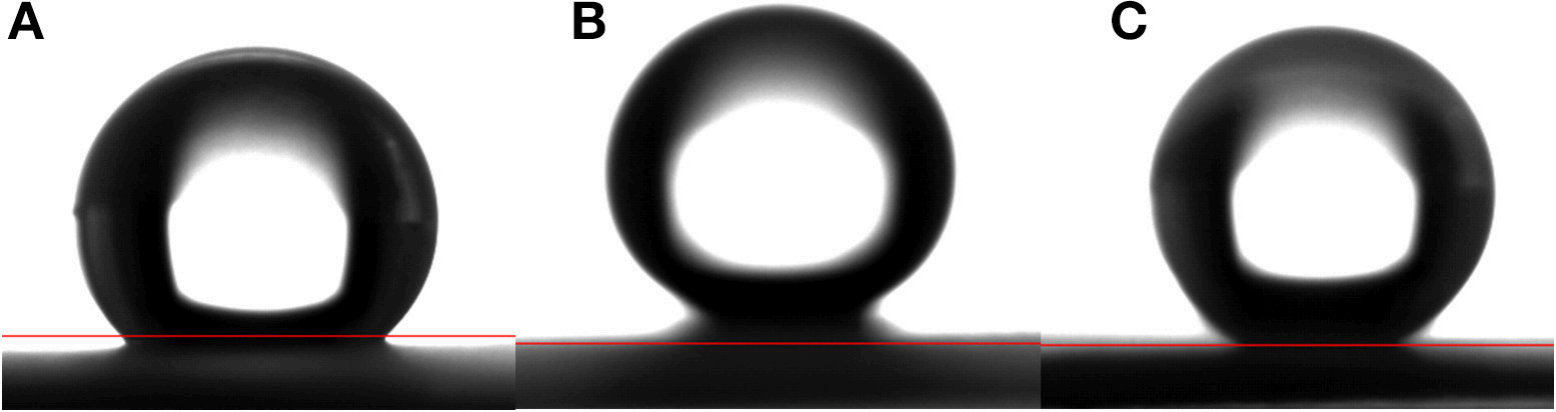
The water contact angle on the ZnO nanorod arrays with different reaction time **(A)** 2 h, **(B)** 4 h, and **(C)** 6 h.

Figure 3 shows the water contact angle on the ZnO nanorod arrays with different reaction time. The water contact angles on ZnO nanorod arrays modified by stearic acid are about 133.3°, 160.5°, and 149.1°, respectively. Wettability of ZnO nanorod arrays can be explained by the Cassie equation (Cassie and Baxter, 1944)

$$\begin{array}{l} \cos\theta^{*}=-1+f_{1}\left(1+\cos\theta \right) \end{array}$$

Where θ is water contact angle of smooth glass, θ^*^ is water contact angle of ZnO nanorod arrays, and *f*_1_ is surface fraction of solid. As shown in Figure 1A, the surface roughness is small because the diameter of ZnO nanorod is small and lodging on the glass substrate. And the water contact angle is smaller. It can be estimated from Figures 1C,E that the *f*_1_ values are about 0.15 and 0.36, respectively. A lot of air can be trapped among ZnO nanorod and acted as a barrier to diminish the contact between water and ZnO nanorod. Based on the above data, the ZnO nanorod arrays with reaction time of 4 h are selected as the research object for the following experiment.

**Figure 4:**
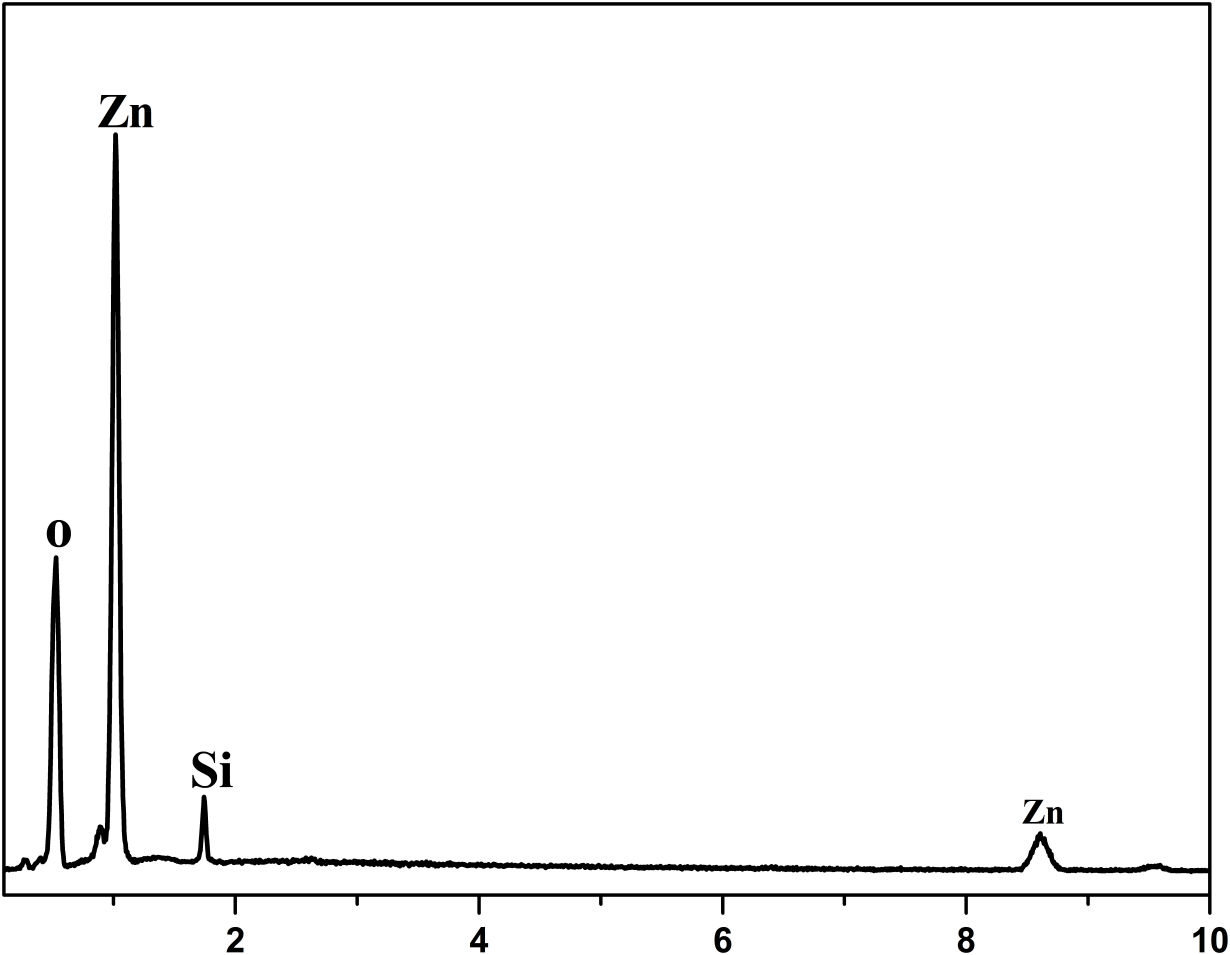
EDS of SiO_2_ shell on ZnO nanorod arrays.

**Figure 5:**
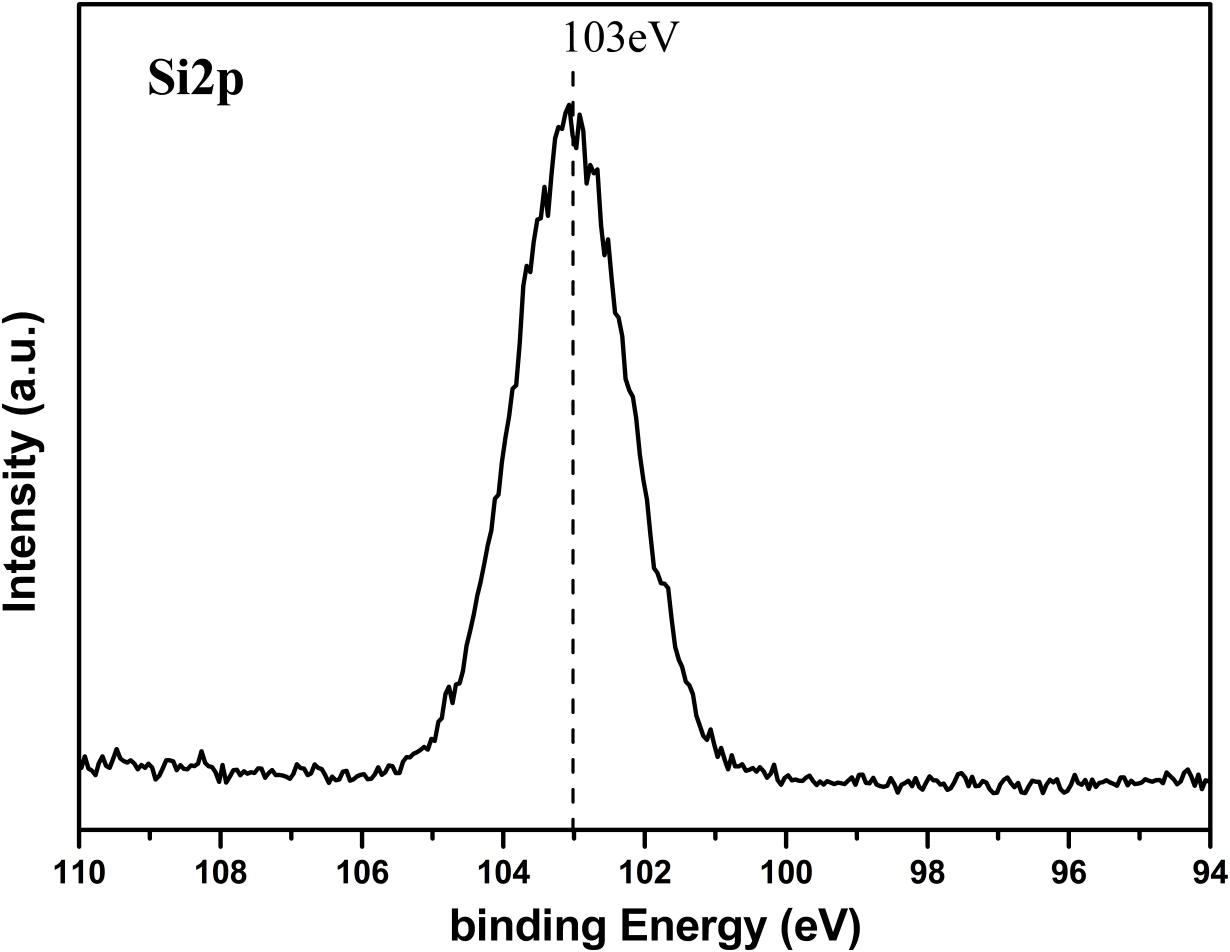
Si2p XPS signal of SiO_2_ shell on ZnO nanorod arrays.

**Figure 6:**
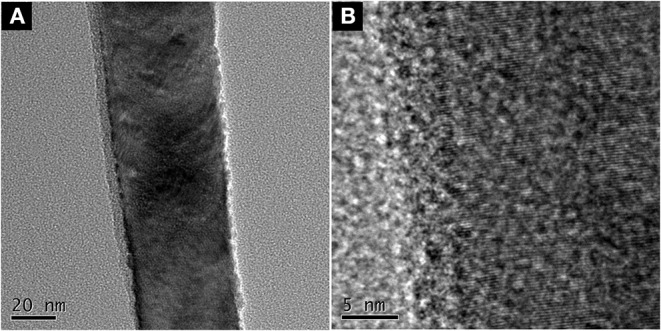
**(A)** TEM image of and **(B)** HRTEM.

ZnO nanorod arrays are coated with a thin SiO_2_ film by pulsed laser deposition (PLD) technique and modified by stearic acid. Surface morphology and wettability on this surface unchanged and are the same as Figure 1 and Figure 3B. This shows that the thickness of SiO_2_ film is so thin that it negligibly changes the morphology of ZnO nanorod arrays. The X-ray diffraction patterns of SiO_2_ shell on ZnO nanorod arrays are the same as Figure 2 and there are no characteristic peaks of SiO_2_. This shows that the amount of SiO_2_ is very small or exists in amorphous state. In order to prove the presence of SiO_2_, the elements analysis of SiO_2_ shell on ZnO nanorod arrays is carried out with energy dispersive spectrum (EDS). Figure 4 exhibits the elements of prepared sample consisted of O, Zn and Si. To judge the deposition film, X-ray photoelectron spectroscopy (XPS) analyses with an Mg Kα X-ray source is used. It can be seen in Figure 5 that the position of Si 2p is 103 eV. This shows that the deposition film is SiO_2_. Figure 6 shows the TEM image of ZnO nanorods. A dense shell of SiO_2_ is observed to coat the surface of ZnO nanorods (Figures 6A,B) and the thickness is about 5 nm. These show that the surface of ZnO nanorods is coated by SiO_2_ film.

**Figure 7:**
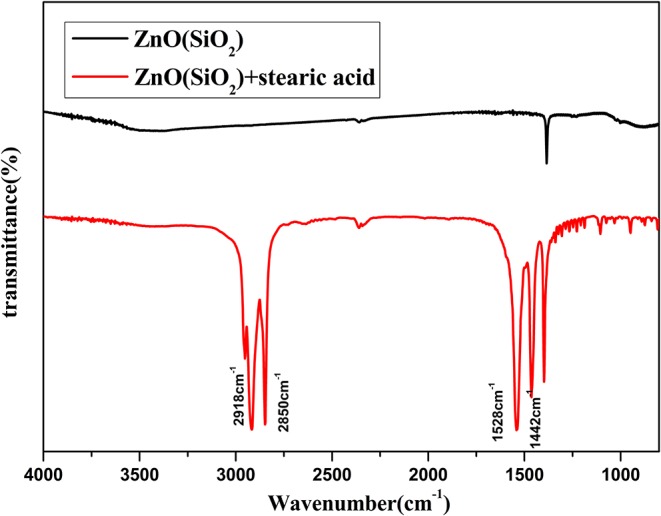
FITR spectra of SiO_2_ shell on ZnO nanorod arrays before and after modification by stearic acid.

Figure 7 illustrates Fourier transform infrared (FTIR) spectra of SiO_2_ coated on ZnO nanorod arrays before and after modification by stearic acid. Two absorption peaks at 2,918 and 2,850 cm^−1^ are the antisymmetric and symmetric stretching vibration of methyl and methylene. Two absorption peaks at 1,528 and 1,442 cm^−1^ are the stretching vibrations of -COOH- in the CH_3_(CH_2_)_16_COO- group (Meth and Sukenik, 2003). This shows that stearic acid has been successfully assembled on the surface of sample.

**Figure 8:**
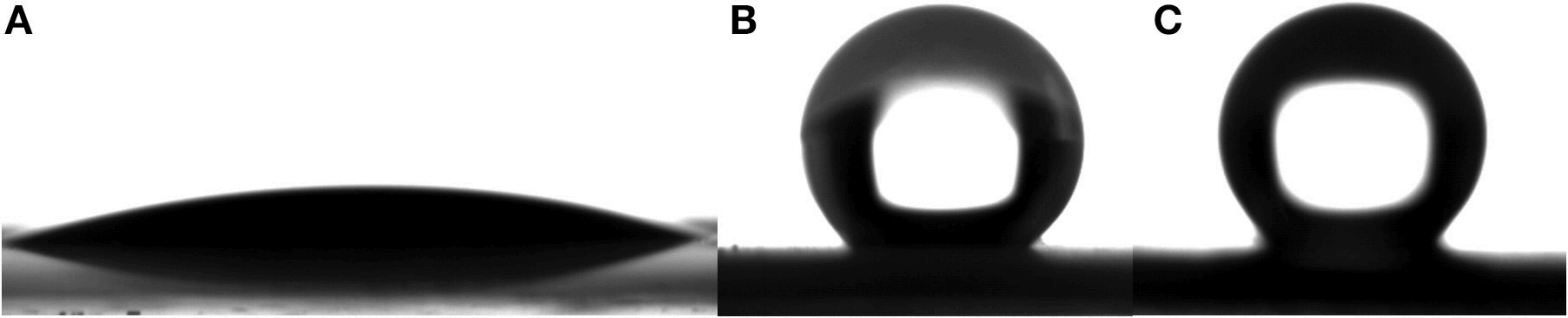
**(A)** is water contact angle on the surface of bare ZnO nanorod arrays after exposure to UV for 2 h. **(B,C)** are water contact angle on the surface of SiO_2_ shell on ZnO nanorod arrays with deposition times 5 and 10 min after exposure to UV for 4 h. All the samples were modified by stearic acid.

ZnO is a kind of material with good photocatalytic properties and has a strong oxidation capacity (Zhang et al., 2004). It is known that photo-generated electron-hole pairs under UV irradiation in ZnO react with oxygen and water, producing hydroxyl radicals (Sun et al., 2001). So it can completely decompose organic compounds modified on the surface of ZnO eventually to carbon dioxide and water. Figure 8A shows that the water contact angle on ZnO nanorod arrays under UV irradiation for 2 h. the contact angle is about 16.1°. This shows that the wettability of ZnO nanorod arrays changes from superhydrophobicity to hydrophilicity. Figures 8B,C shows the water contact angle on SiO_2_/ZnO/glass structure under UV irradiation for 4 h with 5 and 10 min deposition time. The water contact angles are about 143.2° and 160.5°, respectively. This indicates that SiO_2_ can effectively protect the superhydrophobic surface of ZnO nanorod arrays.

**Figure 9:**
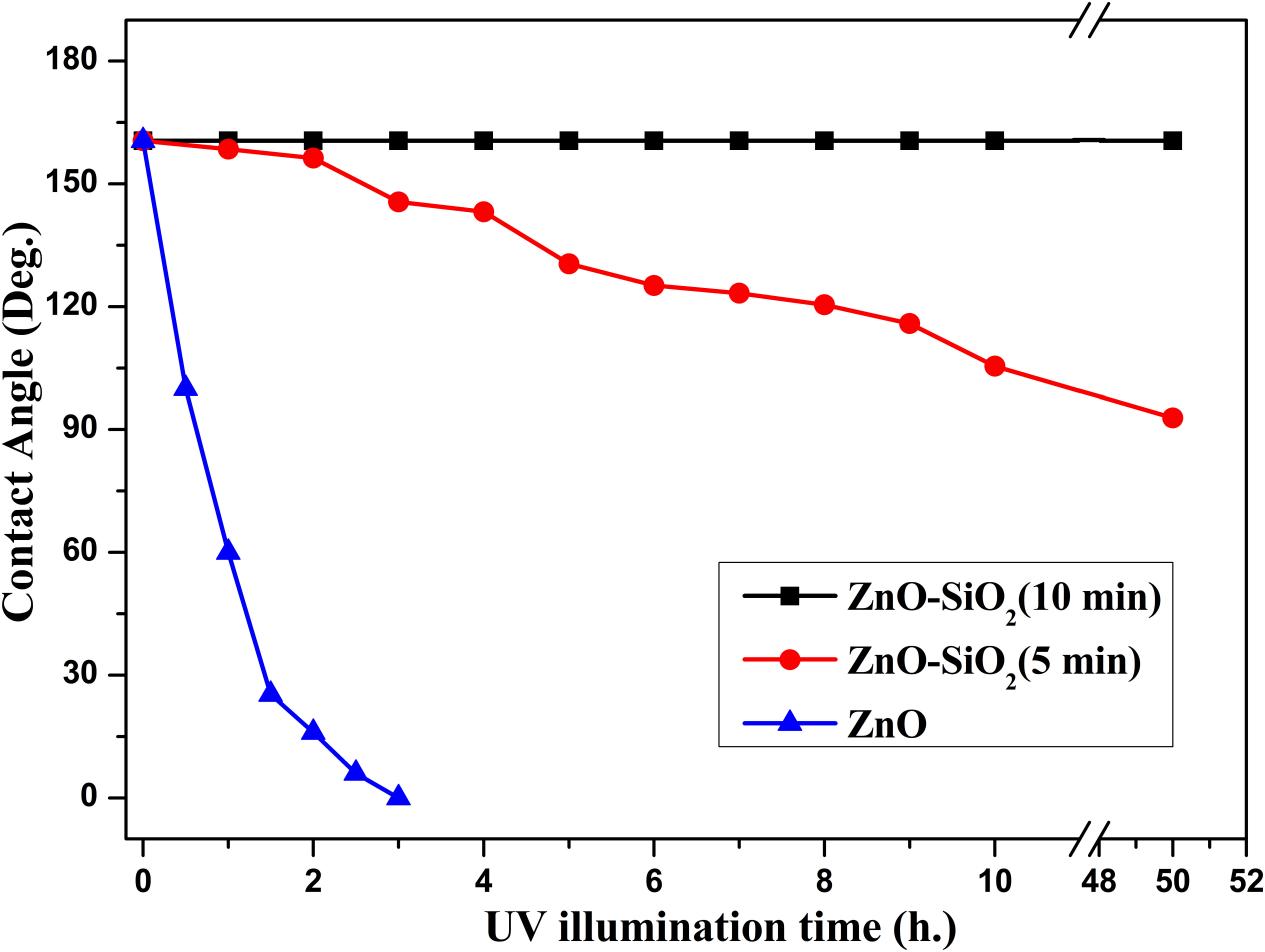
The relationship between deposition time of SiO_2_ shell modified by stearic acid and water contact angle under UV illumination.

**Figure 10:**
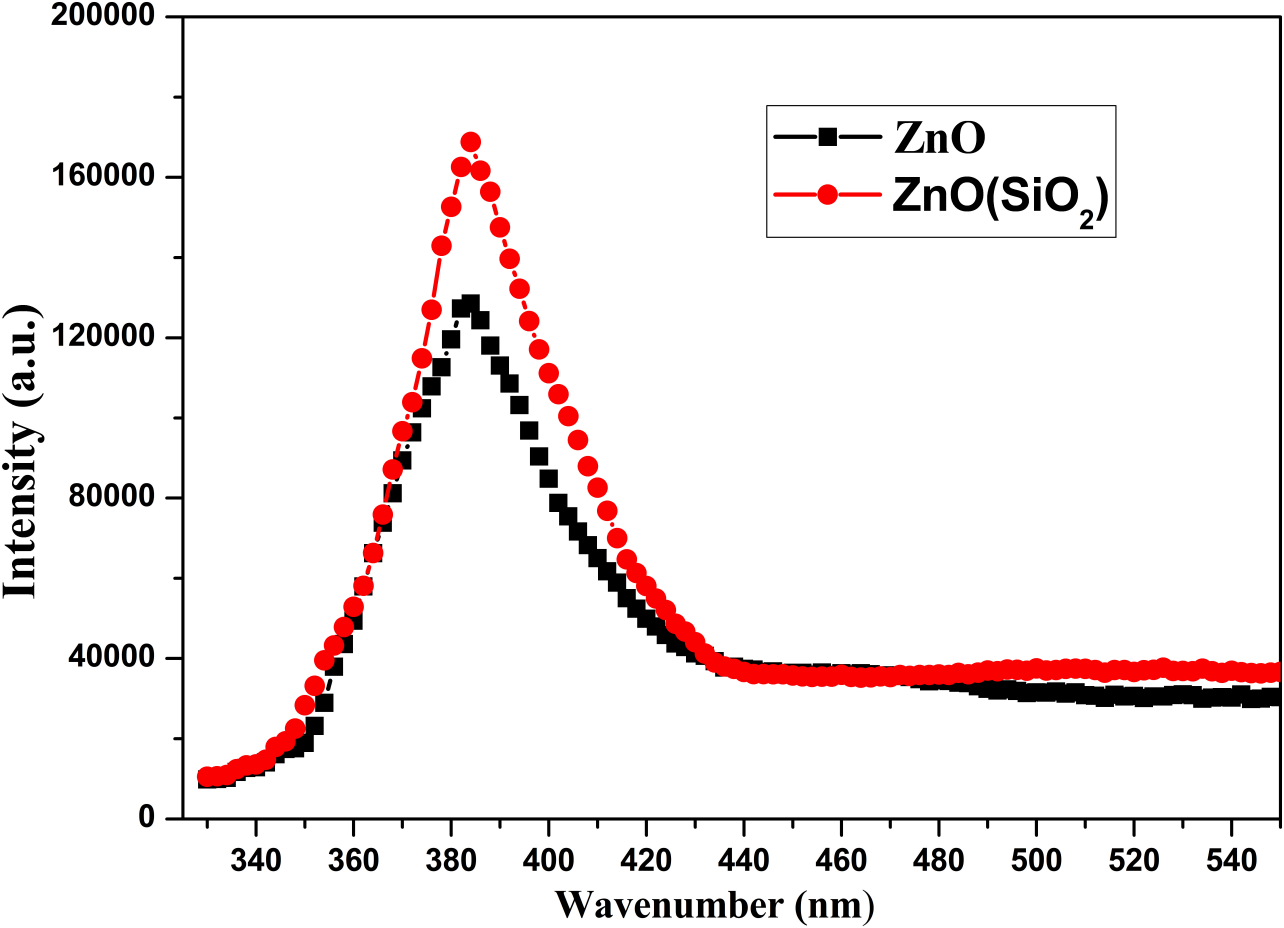
Photoluminescence spectra of bare ZnO and SiO_2_ coated on ZnO with deposition of 10 min.

Figure 9 illustrates the further relationship about the change of the water contact angle on SiO_2_/ZnO/glass structure under UV irradiation. The water contact angle on bare ZnO nanorod arrays decreased quickly to 0° in 3 h. When the deposition time is 5 min, the water contact on SiO_2_/ZnO/glass structure only decreased from 160.5° to 92.8° in 50 h. The water contact even nearly unchanged when the deposition time is extended to 10 min. The valence of SiO_2_ is far lower and conduction band is higher than ZnO, respectively. Electron-hole pairs generated in ZnO are hard to get through barrier between ZnO and SiO_2_, as a result, electron-hole pairs are difficult to react with stearic acid modified on the surface of SiO_2_. This is the primary reason for the formation of UV-durable superhydrophobic surface. Electron-hole pairs will have to recombine with each other to generate photoluminescence through radiative transition, leading to the increase in the intensity of UV emission. The photoluminescence spectra of samples were performed by FLS 920 using excitation of 325 nm. Figure 10 gives the photoluminescence spectra of bare ZnO and SiO_2_ coated on ZnO with deposition of 10 min. It can be proved that the intensity of UV emission of SiO_2_ coated on ZnO is stronger than bare ZnO. In addition, Si-O bonds in SiO_2_ readily react with hydroxyl radicals on the surface of ZnO (Wu et al., 2007), as a result, ultra-thin SiO_2_ shell film deposited through PLD is essential to the formation of UV-durable superhydrophobic surface.

**Figure 11:**
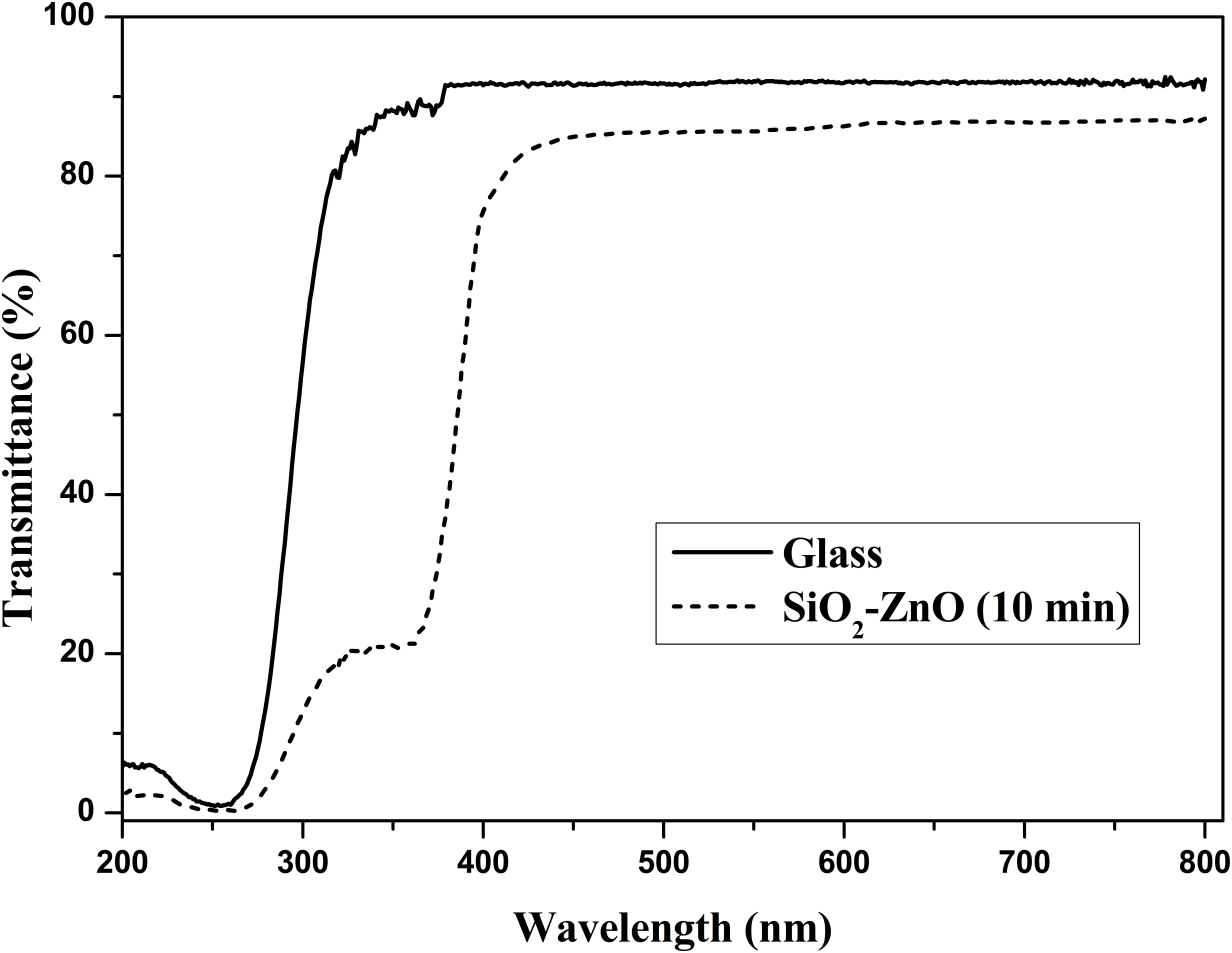
Transmittance spectra of SiO_2_ shell on ZnO nanorod arrays and bare glass.

High transmittance is the basic requirement for the front electrode materials in solar cells. ZnO nanostructure has good transmittance in visible and infrared region due to high band gap of 3.2 eV. The thickness of SiO_2_ film is so thin that the effect on the surface morphology and water contact angle is negligible. Figure 11 gives the transmittance spectra of SiO_2_ shell on ZnO nanorod arrays and bare glass. It can be shown that transmittance is around 85% when wavelength ranges from 400 to 800 nm and with a small transmittance loss compared to bare glass. This loss in transmittance is acceptable compared to a loss of nearly half the efficiency due to dust.

## Conclusion

A large area of vertically aligned ZnO nanorod arrays was prepared through chemical hydrothermal process. Ultra-thin SiO_2_ shell film was deposited on ZnO nanorod arrays through PLD, and subsequently modified by stearic acid. This SiO_2_/ZnO/glass structure exhibited well UV-durable superhydrophobicity and highly transmittance. These properties can have important applications in solar cells.

## Data Availability Statement

The datasets generated for this study are available on request to the corresponding author.

## Author Contributions

HW, QLi, QLiu, XZ and QG participated in the discussion and gave useful suggestions. The manuscript was composed by HL and JZ. All authors read and approved the final manuscript.

### Conflict of Interest

The authors declare that the research was conducted in the absence of any commercial or financial relationships that could be construed as a potential conflict of interest.
